# From Attitude to Behavior: The Effect of Residents’ Food Waste Attitudes on Their Food Waste Behaviors in Shanghai

**DOI:** 10.3390/foods13142201

**Published:** 2024-07-12

**Authors:** Caixia Li, Zhou Zhou, Zengjin Liu, Qiushuang Fang, Guanghua Han

**Affiliations:** 1 Institute of Agricultural Science and Technology Information, Shanghai Academy of Agricultural Sciences, Shanghai 201403, China; 15800718579@163.com (C.L.); zhouzhou10629@163.com (Z.Z.); sahafqs@163.com (Q.F.); 2 China School of International and Public Affairs, Shanghai Jiao Tong University, Shanghai 200240, China; hanguanghua@sjtu.edu.cn

**Keywords:** food waste attitude, food waste behavior, multivariate ordered probit model, moderating effect

## Abstract

Preventing food waste is important. Analyzing the effects of food waste attitudes on urban residents’ waste production behaviors is necessary to reduce food waste. As Shanghai is a mega-city with a population of 24 million people, once food is adequately supplied, more attention is paid to the safety of food in terms of quantity, quality, and nutrition. COVID-19 gave a shock to the food supply in Shanghai, which in turn resulted in food waste behavior. The moderating effect of pandemic during the COVID-19 is one that has rarely been mentioned in previous studies. An investigation of urban residents was conducted in Shanghai. A total of 1030 valid questionnaires were collected in October 2020. This study examined the influence of food waste attitudes on food waste behavior under the moderating effects of pandemic expectations, quantity safety, quality safety, and price stability using a multivariate ordered probit model. The results show that food waste attitudes had a significant negative effect on food waste behavior at a *p* < 0.01 level of significance, which means that the more people feel shame about food waste, the less food waste they will have. The interaction coefficient between food waste attitudes and pandemic expectations was positive at a *p* < 0.1 level of significance. This paper concludes with strategies for dealing with food waste in the future.

## 1. Introduction

In recent years, with the accelerated development of urbanization and improvement of economic standards, an increasing number of people have begun to realize the serious problem of food waste [1]. Food waste is not only a waste of resources but also a sign of environmental damage and social injustice [2]. Although an increasing number of urban residents have begun to focus on and take action to reduce food waste, many people remain indifferent to this problem [3]. A new perspective on food waste under the conditions of the current health crisis and anticipated economic recession is required [4]. Therefore, examining the effect of food waste attitudes on food waste behavior among urban residents is required to develop effective policies and action plans to reduce food waste. According to the Shanghai Municipal Bureau of Statistics, 10,479,652 of Shanghai’s residents are from other provinces and cities, accounting for 42.1% of the population [5]. This suggests the prevalence of food waste in the study of Shanghai. As one of the largest cities in China, Shanghai has a high population density and a large urban scale. Moreover, as an international metropolis, Shanghai has a high level of economic development, and people have high requirements in terms of living standards and lifestyles. This makes Shanghai representative of the food waste problem during the COVID-19 pandemic, and the results of this study are generalizable. Therefore, the food waste problem in Shanghai may have been challenging during the pandemic [6]. This has implications for food waste reduction countermeasures at the national level.

According to the Food and Agriculture Group of the United Nations, one-third of globally produced food is wasted or depleted [7]. Therefore, end-consumption food waste in China cannot be ignored by policy makers. According to a 2015 survey conducted by the Chinese Academy of Sciences on the catering industry in four cities (Beijing, Shanghai, Chengdu, and Lhasa), the food waste rate in the catering industry was 12% [8]. That is a very high rate for China. This not only affects the economy, society, and the environment but also poses a risk to food security in China [9]. Due to the COVID-19 pandemic, the world is facing unprecedented food security challenges. To understand the global food situation, scholars have analyzed the food supply and food security coping strategies in China and globally and have proposed strategies to ensure China’s food security [10]. Global food production is generally stable, in line with expectations, and has a strong resistance to shocks [10]. Although China’s supply of major foodstuffs is secure, imported commodities such as soybeans were affected before COVID-19 [11]. The COVID-19 pandemic has led to production disruptions and income losses, in addition to poor public health and mortality [12]. A serious consequence of the pandemic for welfare is the reduction in food access [12].

However, some studies have identified a decrease in food waste during the COVID-19 pandemic, with people being cautious about food purchases and consumption [13,14,15,16]. Therefore, researchers have called for measures to enhance the stability and adaptability of food supply chains and to reduce waste production. No studies have been conducted on the moderating effect of pandemic expectations on food waste behavior. This study aimed to systematically investigate the impact of food waste attitudes on the waste production behavior of urban dwellers in Shanghai during the COVID-19 pandemic and provide policy recommendations. Moreover, this study examined the influence of individuals’ socioeconomic status, family environment, and educational background on food waste attitudes and behaviors.

## 2. Literature Review and Theoretical Analysis

### 2.1. Literature Review

Many scholars were interested in examining food waste reduction before the COVID-19 pandemic. The factors that influence food waste are complex and multifaceted. For instance, a study found that few habitual and emotional variables were important determinants of the participants’ willingness to reduce food waste and their current food waste behaviors [17]. Relationships have been identified between personal habits, attitudes, addiction to marketing, sales strategies, and food waste behavior [18]. Family composition and habits were major contributors to food waste [19]. A study showed that four main attitudes were most likely to contribute to food waste behavior: fussy (wasting food because it does not smell or look good); forgetful (forgetting there is food in the fridge or on the shelves); thrifty (tending to skip fruits and vegetables and striving to waste little); and exaggerated (over-buying or over-cooking) [20]. However, among university students, the problem of food waste is insidious, and it is feasible to understand consumer food waste behavior by focusing on their practices, routines, and habits [21]. Food waste occurs at all stages of the food supply chain, and private households have been identified as major players in generating food waste [21]. For instance, changes in eating habits and food surpluses at certain times of the year have significant impacts on food waste behavior. [22]. Numerous studies have shown that the factors influencing food waste behavior are complex and diverse.

The COVID-19 pandemic caused serious repercussions worldwide. During lockdowns associated with the COVID-19 pandemic, four new concepts of food consumption and food waste habits emerged at the household level [23]. Waste avoidance and cost-effective procurement behavior during the COVID-19 pandemic were the most important food waste avoidance factors, followed by procurement planning, knowledge and use of labelling information, food storage, and cooking skills [24]. An online survey of 1959 adult respondents showed that people in areas heavily affected by the pandemic had a clear understanding of food waste in their households, were careful in preparing and purchasing food, and were strongly influenced by the pandemic to change their behavior [25]. Further analyses showed that food-waste-related thoughts and behaviors differed significantly by socio-demographic characteristics such as gender, household size, and employment status. This study also showed that the COVID-19 pandemic contributed to improvements in attitudes and behaviors towards food, such as concern about food waste, efforts to reduce food waste, and attempts to cook at home [25]. As food delivery apps (FDAs) have become prominent sources of ordering food during the COVID-19 pandemic, one study on the role of FDAs in food waste generation revealed a positive association between trust, price advantage, and attitude [26]. Moreover, intentions to reduce waste, routines for purchasing food, and routines for managing leftovers or uneaten food were positively associated with the economic value of reducing food waste [27]. The results of a study on food waste behavior in Italy during the pandemic illustrated that consumers who were forced to stay at home for 24 h a day were likely to perceive food waste and reduce its amount, whereas discontinuous smart working makes food purchases necessary [28]. Conversely, in Serbia, food waste increased during the COVID-19 pandemic, as consumers reduced the number of shopping trips and bought more than usual during the pandemic [29]. This provides evidence of the impact of COVID-19 on food waste in various countries. Therefore, examining food waste behavior during the COVID-19 pandemic is worthwhile. The results could provide a reliable basis for policymakers.

### 2.2. Theoretical Analysis

Food waste is a major health concern. This study examined food waste attitudes as the core influencing factor affecting food waste behavior. The concept of food waste and its estimation methods have also been extensively investigated [30,31]. A previous study used behavioral reasoning theory (BRT) to explore the factors influencing consumers’ motivations and the barriers to purchasing optimal foods [32]. In addition, a survey of 244 Romanian consumers examined the influence of intentions to not waste food, and the results showed that consumer planning and shopping routines were important predictors of food waste [33]. Food waste may emerge from the interaction of activities associated with the planning, shopping, storage, preparation, and consumption of food [34]. Some studies have examined the severity of food waste behavior in terms of household income, food packaging, and kitchen waste [35,36,37].

In addition, the impact of artifacts, which can lead to a downward bias in the observed effect sizes, should be minimized, leading to implications for the use of moderators [38]. The traditional definition of effect size has been questioned. Consequently, studies have described general models for simultaneously estimating mediating and moderating effects, illustrated the utility of combining these effects into a single model, explained the possible correlation of the effects in the model, and assessed the statistical methods for these effects [39]. Above all, there is a significant effect of food waste attitudes on food waste behavior. 

Thus, we proposed the following hypothesis:

**H1.**  *Food waste attitude has a significant negative effect on food waste behavior.*

Food waste is the result of consumers’ behavioral choices, and consumer behavior is largely influenced by descriptive norms [40]. The consumption chain is an important and growing component of food waste [41]. In addition, gender, cost of living, basic attitudes toward food waste, food taste evaluation, participation in the “empty plate” action, and weight perception have a significant effect on the frequency and proportion of food waste in college student cafeterias [42]. Previous studies have shown that subjective norms and perceived behavioral control to reduce food waste intentions had a significant positive influence, whereas the effect of attitude was not significant [43,44,45]. Moreover, a questionnaire showed that participants tended to overestimate others’ food-wasting behaviors and approval of wasteful behaviors, both of which had a significant positive impact on food waste [46]. This implies that these misperceptions contributed to people’s food-wasting behaviors [46]. For instance, the amount of food left over at dinners with friends and other public settings is significantly higher than that of ordinary daily meals, with over-ordering and unmatched tastes being the major reasons for leftovers [3,47,48]. Consumers with lower education and ages are more likely to waste food [3,47,48]. In the global resource scarcity environment, attitudes toward food waste are particularly important, and food waste is influenced by all aspects of life [49]. Moreover, the perceived behavioral control and routines related to shopping and the reuse of leftovers are the main causes of food waste, while planning routines are an indirect cause [50]. Furthermore, perceived behavioral control and good provider status are significantly correlated with the amount of waste in most food categories [51].

Pandemic expectations, quantity safety, quality safety, and price stability serve as regulatory variables in food waste behavior, and the regulatory effect may play an important role in prompting people to reduce waste behaviors. The moderating effect model is widely used in various disciplines, such as economics and psychology [52]. Previous studies have applied the moderating effects model to various fields while deriving new estimation methods [53,54,55]. Moreover, food-related personality traits, such as food neophobia and involvement, moderated consumers’ organic food choice behaviors [56]. Another study demonstrated that self-efficacy mediated the relationship between perceived collective efficacy and willingness to reduce food waste [57]. However, studies on the moderating effect of pandemic expectations on the relationship between food waste attitudes and food waste behavior in Shanghai are lacking. This study selected four non-human factors—pandemic expectations, quantity safety, quality safety, and price stability—as moderating variables in the relationship between food waste attitudes and food waste behavior, reducing the influence of human factors.

Thus, we proposed the following hypothesis:

**H2.**  *Pandemic expectations, quantity safety, quality safety, and price stability moderate the effect of food waste attitudes on food waste behavior, with pandemic expectations having the most significant impact.*

The impact of public health events on tourists’ food waste behavior during the COVID-19 pandemic deserves attention [58]. China’s food supply and demand have been tightly balanced for a long time, and the problem of food waste in the consumption link is prominent [59]. The COVID-19 pandemic created anomalies in the international food supply chain, and China must take precautions to protect the food security of 1.4 billion people [60]. The 2020 COVID-19 outbreak was during spring planting in China, which affected food production [61]. Moreover, the further deterioration of the US–China relationship limited imports of food to China from the United States [61]. This also provides a partial reference for people’s expectations of the pandemic, making the moderating variable of pandemic expectations more objective. Therefore, stabilizing food production, food prices, and social expectations guarantees China’s food security in the pandemic era [62] and may play a role in reducing food waste behavior. In addition, food waste can be affected by inappropriate storage or short shelf life [63].

Common attitudes toward food waste can directly contribute to food waste behavior. Studies have shown that people waste more food during a pandemic than in regular situations. This suggests the presence of a moderating effect of pandemic expectations.

**Figure 1:**
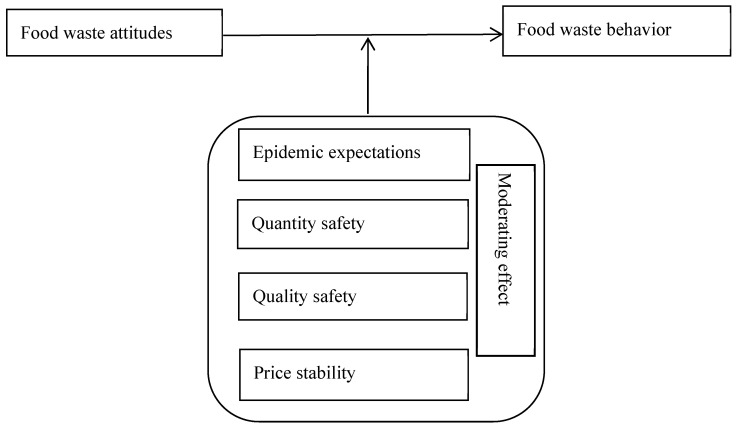
Theoretical analysis framework.

A theoretical analysis framework for the moderating effect of food waste attitudes and food safety status on food waste behavior during the pandemic is presented in Figure 1.

## 3. Data Source and Methodology

### 3.1. Data Source

We conducted a questionnaire survey of urban residents in Shanghai during the COVID-19 pandemic, with 1030 valid questionnaires collected in October 2020. We sampled 15 districts in Shanghai, including Huangpu, Xuhui, Changning, Jing’an, Putuo, Hongkou, Yangpu, Minhang, Baoshan, Jiading, Jinshan, Songjiang, Qingpu, Fengxian, and Pudong New Area, except Chongming. This investigation was conducted primarily among consumers of agricultural products. Investigation locations included farmers’ markets, supermarkets, etc. The survey team consisted of professors and students from the Shanghai Ocean University and the Shanghai Academy of Agricultural Sciences. The investigators were dispersed in various districts of Shanghai and conducted one-on-one interviews with randomly selected people to ensure the recovery rate and accuracy of the survey questionnaire. We had a total of 1141 respondents. Despite the relative easing of the epidemic in October 2020, a small percentage of people did not want to be interviewed face-to-face. We interviewed face-to-face and achieved a 90.27% return rate on the questionnaire. Due to the high return rate and the high coverage rate of the questionnaire, our sample is representative.

### 3.2. Model Settings

As food waste behavior is a multivariate ordered variable, a multivariate ordered-probit model was used for the estimation. To test the impact of food waste attitudes on food waste behavior, the following model was established:

(1) $$Y=\alpha_{0}+\alpha_{1}Ati+\alpha_{2}Exp+\alpha_{3}Sup+\alpha_{4}Qua+\alpha_{5}Pri+\sum \beta_{i}X_{i}+\varepsilon_{1}$$

To estimate the moderating effects of pandemic expectation, quantity safety, quality safety, and price stability on the influence of food waste attitudes on food waste behavior, a regression model, including the interaction terms of food waste attitudes and pandemic expectation, food waste attitudes and quantity safety, food waste attitudes and quality safety, and food waste attitudes and price stability, was established based on Equation (1) as follows:

(2) $$Y=\alpha_{0}+\alpha_{1}Ati+\alpha_{2}Exp+\alpha_{3}Sup+\alpha_{4}Qua+\alpha_{5}Pri+\alpha_{6}Ati\times Exp+\alpha_{7}Ati\times Sup+\alpha_{8}Ati\times Qua+\alpha_{9}Ati\times Pri+\sum \beta_{i}X_{i}+\varepsilon_{2}$$

In Equations (1) and (2), Y represents food waste behavior, Ati represents food waste attitudes, Exp represents pandemic expectations, Sup represents the degree of confidence in quantity safety, Qua represents the degree of confidence in the quality safety of agricultural products, Pri represents the degree of stability of local agricultural product prices, Ati×Exp is the interaction term between food waste attitudes and pandemic expectation, Ati×Sup represents the interaction between food-waste attitudes and quantity safety, Ati×Qua is the interaction term between food waste attitudes and quality safety, and Ati×Pri is the interaction term between food waste attitudes and local agricultural product price stability. $X_{i}$ comprises a series of control variables. In addition, $\alpha_{1},\alpha_{2},\alpha_{3},\alpha_{4},\alpha_{5},\alpha_{6},\alpha_{7},\alpha_{8}$, and $\alpha_{9}$ are food waste attitudes, pandemic expectations, quantity safety, quality safety, local agricultural product price stability, and the estimated coefficients of each interaction term, respectively. $\alpha_{0}$ is a constant term; $\varepsilon_{1}$ and $\varepsilon_{2}$ are random error terms.

### 3.3. Analytical Methods

A multivariate ordered-probit model was applied to verify the effects of attitudes toward FW on food waste behavior (Model 1). Model 2 examined the effects of pandemic expectations, quantity security, quality security, and price stability on food waste behavior. Model 3 explored the effect of food waste attitudes and pandemic expectations, food waste attitudes and quantity safety, food waste attitudes and quality safety, and food waste attitudes and price stability on food waste behavior. A comprehensive analysis using the chi-squared test (LR chi-squared) of Models 1, 2, and 3 indicated that the regression models were well-fitted, and all were significant at the 1% level. Therefore, the results are valid.

Furthermore, we applied an interaction term to verify whether the concerns about pandemic expectations, security of supply, and the price of agricultural products moderated the influence of food waste attitudes on food waste behavior. Model 4 added four interaction terms based on Model 3: pandemic expectations, quantity safety, quality safety, and price stability.

Moreover, to test whether the model was affected by outliers, non-normal distributions, and other factors, we conducted a robustness test to a multivariate ordered probit model using RStudio.

## 4. Results

### 4.1. Participant Characteristics

The participant characteristics are presented in Table 1. As mentioned in Section 3.1, the sample distribution was balanced, which reduced the model estimation error.

### 4.2. Variable Analysis and Descriptive Statistics

Table 2 presents the statistics and descriptions of the variables. The results demonstrate that the severity of food waste was skewed toward not wasting, and the attitude toward waste tended to be within the acceptable range. Moreover, respondents concerned about the severity of future pandemics were skewed, whereas quantity safety, quality safety, and price safety tended to be stable.

This paper focuses on the moderating effects of pandemic expectations, quantity safety, quality safety, and price stability on food waste attitudes affecting food waste behavior. To verify the reliability of the scales in the questionnaire, a reliability analysis was performed (Table 3).

The Cronbach’s alpha coefficient was estimated to be 0.71 for reliability. Combined with the Cronbach’s alpha coefficients for each variable after the deletion of items in Table 3, we concluded that the questionnaire scale is reliable.

### 4.3. The Impact of Food Waste Attitudes on Food Waste Behavior

The results demonstrate that the level of significance of each variable in the probit model remained the same, indicating that the estimation results of the model are robust. Food waste attitudes had a significant negative impact on food waste (Models 1 and 3). In other words, feeling ashamed about waste and advocating frugality were associated with a focus on reducing food waste. In addition, concern about future pandemic expectations had a significantly positive impact on food waste (Models 1 and 3). The degree of confidence in food supply security had a significant positive impact on food waste (Model 4 in Table 4). However, the degree of confidence in the quality and safety of agricultural products and the stability of agricultural product prices had no significant impact on food waste.

As shown in Table 3, the necessity of saving food during the pandemic had a significant negative impact on food waste. Gender and age had significant negative impacts on food waste. Men wasted less food than women, and older people were more inclined to reduce food waste than younger people. In addition, family income had a significant positive impact on food waste. Finally, household registration, education level, family size, number of children in the family, and number of older people in the family had no significant impact on food waste.

### 4.4. Pandemic Expectations and Regulatory Effects of Agricultural Product Safety

Pandemic expectations and agricultural product safety, including both supply and price, moderated the influence of food waste attitudes on food waste behavior. As shown in Model 4, the effect of food waste attitudes on food waste behavior was positive but not significant. The interaction coefficients between food waste attitudes and pandemic expectations, between food waste attitudes and quantity safety, and between food waste attitudes and quality safety were significantly negative. Conversely, the interaction coefficient between food waste attitudes and price stability was significantly positive. This reveals a significant moderating effect of pandemic expectations and food supply and price security on the relationship between food waste attitudes and food waste behavior. In addition, the negative interaction coefficients of food waste attitudes with pandemic expectations, quantity safety, and quality safety indicated that confidence about agricultural product supply security and quality increased as the degree of concern about pandemic expectations increased. Moreover, confidence about agricultural product quality reduced the positive effect of food waste attitudes on food waste behavior. Furthermore, price stability of local agricultural products strengthened the positive effect of food waste attitudes on food waste behavior.

### 4.5. Robustness Test

The robustness test results indicate that the residents’ attitudes to food waste were positively significant (Table 5). In addition, future pandemic expectations, confidence about the safety of agricultural products, advocating food conservation during pandemics, and household income were significant. The interaction coefficients between food waste attitude and pandemic expectations and between food waste attitudes and confidence in food safety were significant. The coefficients of the core independent variables and significant variables of confidence about food safety, advocating food conservation during pandemics, and household income were positive. Conversely, the cross-term coefficient of attitudes toward food waste and confidence about food safety was negative.

A comparison of the statistical results of the multivariate ordered-probit model reveals that the significance of the variables and the positive and negative coefficients coincided with each other, except for the factors influencing the necessity of advocating for food conservation during pandemics. This indicated that the proposed model was robust. The influence of advocating for food conservation during the pandemic on food waste changed from negative to positive after the robustness test, which may be due to the presence of a small number of outliers. However, the statistical analysis results are not significantly affected by the outliers, indicating that the results of the model have a high degree of resistance to interference and that the outliers had a small impact on the analysis results. Thus, the model results have a high degree of reliability; that is, the variables explained food waste behavior.

Above all, the effect of food waste attitudes on food waste behavior changed from negative to positive after adding pandemic expectation as a moderating variable. Therefore, the moderating variable of pandemic expectation is significantly positive. The above results indicate that our research hypothesis was verified, i.e., the hypothesis was valid.

## 5. Conclusions

This study conducted a questionnaire survey of food waste in Shanghai during the COVID-19 pandemic. Food waste attitude positively affected food waste behavior. Moreover, confidence in the safety of agricultural products and household income positively affected food waste behavior. This study demonstrated that the COVID-19 pandemic affected agricultural waste. However, factors such as embargo measures, transportation restrictions, and economic instability may have led to increased stagnation and the waste of agricultural products [64]. The problem of consumer food waste practices is complex and involves both sociocultural and material factors [65,66]. Although previous studies have examined the impact of food waste attitudes on food waste behavior during epidemics, they have not explicitly presented a positive or negative picture of the moderating role of people’s epidemic expectations in the impact of food waste attitudes on food waste behavior.

Our results reveal two significant aspects regarding food waste. First, a large amount of food waste was observed during the COVID-19 pandemic. This may be because the fear of future pandemics may cause people to hoard food, exceeding their normal food needs on weekdays and resulting in waste. Consumer-generated food waste is the main source of food loss and waste [67]. The last kilometer of food security faces many challenges, and waste in food consumption remains significant [68]. Although several advocacy provisions for food conservation exist in China’s laws and regulations, they do not specify food waste offenses and lack a systematic structure and enforceability [69,70] This makes providing targeted punishment for specific food waste offenses challenging [69,70]. Reducing food waste on the consumer side is required to ensure food security. Second, public awareness of reducing food waste is poor. China’s food supply has long been sufficient and stable; however, demographic, and environmental factors continue to challenge this supply, and the annual amount of food loss and waste in China is staggering [71]. In metropolitan cities such as Shanghai, where consumption levels are higher than those in other provinces and cities in China, people are indifferent to food waste.

Our results suggest that the government should take measures to encourage urban residents to reduce the wasteful behavior of agricultural products by strengthening publicity about and education on food waste and raising urban residents’ awareness of food waste. In addition, the government should provide convenient facilities to help residents dispose of leftover food and reduce the extent of food waste by strengthening resources for recycling and reuse. Furthermore, our findings suggest that food waste behavior must be controlled to ensure stable prices of agricultural products. Strengthening and improving the Anti-Food Waste Law would inform people that wasting food is undesirable and legally restrict food waste. In addition, the government can learn from the Japanese Consumer Affairs Agency’s annual questionnaire survey on consumers’ awareness of anti-food waste and the local government’s measures to minimize food loss. The government could conduct the Public Opinion Survey on Eating Habits to monitor consumer survey results. The relevant authorities should understand consumer wishes and evaluate the outcome of efforts to reduce food loss and waste [72]. In the UK, six national surveys conducted over 21 months (2014–2015) tracked customers’ self-reported food waste. The results show that combined communication channels and repeated messages over time significantly reduced customer food waste [73]. We can learn and improve this approach to make it more applicable to reducing food waste in Shanghai and all major cities in China. Therefore, measures should be taken to publicize firms’ resistance to food waste and their food waste behaviors using a two-pronged approach, online and offline, with online promotional videos and offline promotional brochures. The perversely wasteful behavior of people during the epidemic is noteworthy. Further research is required to understand the impact of the pandemic on food waste.

We also have a bit of an outlook for the rest of the study. The period in which our study was carried out, October 2020, was a special period. Now that people’s productive lives have returned to normal levels, has food waste behavior changed from the epidemic period? This is what we are going to be looking at in our next study.

## Figures and Tables

**Table 1:** Participant characteristics.

Project	Options	Proportion
Gender	Male	55.24%
	Female	44.76%
Age	Under 20 years old	10.97%
	20–39 years old	73.40%
	40–59 years old	13.30%
	Over 60 years old	6.89%
Household registration	Local	47.38%
	out of town	52.62%
Education level	Elementary school or below	1.17%
	Junior high school	7.28%
	High school	11.65%
	College	15.15%
	Undergraduate	44.27%
	Postgraduate	30.10%
Monthly income (1000 yuan)	Within 10	32.43%
	10–30	47.57%
	40–50	17.38%
	60–100	12.33%
	110–150	0.00%
	More than 150	0.00%

**Table 2:** Variable descriptions and descriptive statistics.

Variable Name	Variable Setting and Assignment	Mean	Standard Deviation
Severity of food waste	Never waste = 1; Basically, no waste = 2; Some waste = 3; Severe waste = 4; Very serious waste = 5	2.17	0.72
Attitudes to food waste	It doesn’t matter = 1; I spent the money myself and dealt with it as I liked = 2; A small amount of waste is within the acceptable range = 3; It’s a shameful waste = 4	3.32	0.70
Expectations for future epidemics (epidemic expectations)	Worried = 1; Not worried = 0	0.64	0.48
Confidence in the supply of agricultural products in Shanghai’s local market (quantity safety)	Reassured = 1; Not reassured = 0	0.88	0.32
Confidence in the safety of agricultural products in the Shanghai local market (quality safety)	Reassured = 1; Not reassured = 0	0.88	0.32
Price stability of agricultural products in the location (price stability)	Stable = 1; Unstable = 0	0.73	0.44
Promoting the necessity of saving food during the epidemic	Not necessary at all = 1; Not very necessary = 2; Average = 3; Somewhat necessary = 4; Very necessary = 5	0.44	0.88
Gender	Male = 1; Female = 0	0.50	0.50
Age	Actual age (years)	32.94	13.51
Household registration	Local = 1; Foreign = 2	1.57	0.50
Educational qualifications	Primary school and below = 1; Junior high school = 2; Technical secondary school/high school = 3; College = 4; Undergraduate = 5; Postgraduate = 6	4.68	1.23
Family size	Actual number of family members (people)	3.38	1.34
Children	Number of children in the family (person)	1.69	0.46
Elderly people	Number of elderly people in the family (person)	0.88	0.44
Household income	Less than 10,000 = 1; 10,000–30,000 = 2; 40,000–50,000 = 3; 60,000–100,000 = 4; 110,000–150,000 = 5; 150,000 and above = 6	2.62	1.65

**Table 3:** Reliability statistics if item is deleted.

Variable	Scale Mean	Scale Variance	Corrected Item-Total Correlation	Cronbach Alpha
Food waste attitude	13.459	9.561	0.174	0.751
Epidemic expectations	13.868	7.101	0.385	0.708
Quantity safety	13.107	6.595	0.674	0.574
Quality safety	13.109	6.647	0.667	0.578
Price stability	13.566	7.154	0.478	0.658

**Table 4:** Impact and moderating effect estimation results of food waste attitude on food waste behavior.

Variable	Model 1	Model 2	Model 3	Model 4
	Coefficient	Coefficient	Coefficient	Coefficient
Constant term				
Food waste attitude	−0.23 ***		−0.24 ***	0.19
Epidemic expectations		0.16 **	0.15 **	0.30
Quantity safety		0.19	0.24 *	1.55 ***
Quality safety		−0.12	−0.07	0.84
Price stability		0.05	0.05	−0.62
Saving food necessity	−0.18 ***	−0.23 ***	−0.18 ***	−0.16 ***
Gender	−0.15 **	−0.13 **	−0.14 **	−0.12 *
Age	−0.01 ***	−0.01 ***	−0.01 ***	−0.01 ***
Household registration	0.07	0.06	0.07	0.07
Educational qualifications	0.04	0.03	0.03	0.04
Family size	−0.02	−0.01	−0.01	−0.02
Children	−0.07	−0.05	−0.06	−0.08
Elderly people	−0.02	−0.02	−0.01	−0.01
Household income	0.05 **	0.05 **	0.04 **	0.04 **
Pandemic expectation moderating effect				−0.05
Quantitative safety moderating effect				−0.42 **
Quality safety moderating effect				−0.30 *
Price stability moderating effect				0.20 *
Log likelihood	−1120.734	−1128.599	−1117.117	−1104.134
Pseudo goodness of fit (Pseudo R-squared)	0.04	0.03	0.04	0.05
LR statistics	95.11950 ***	80.39844 ***	102.3539 ***	128.3202 ***
Prob (LR statistical)	0.000	0	0.000	0.000

Note: ***, **, and * indicate significance at the 1%, 5%, and 10% levels, respectively.

**Table 5:** Robustness test results for the impact on and moderating effects of food waste attitudes on food waste behavior.

Variable	Coefficient	Robust Standard Error
Constant term	1.27 ***	0.38
Attitudes to food waste	0.24 **	0.10
Epidemic expectations	−0.19 *	0.10
Quantity safety	0.20	0.15
Quality safety	0.40 ***	0.15
Price stability	−0.11	0.10
Saving food necessity	0.11 ***	0.02
Gender	0.06	0.04
Age	−0.01 ***	0.002
Household registration	0.03	0.04
Educational qualifications	0.01	0.02
Family size	0.004	0.01
Children	−0.02	0.05
Elderly people	0.02	0.05
Household income	0.03 **	0.01
Pandemic expectation moderating effect	0.05 *	0.03
Quantitative safety moderating effect	−0.06	0.04
Quality safety moderating effect	−0.12 ***	0.04
Price stability moderating effect	0.04	0.03

Note: ***, **, and * indicate significance at the 1%, 5%, and 10% levels, respectively.

## Data Availability

The original contributions presented in the study are included in the article, further inquiries can be directed to the corresponding author.
